# The Infant-Derived *Bifidobacterium bifidum* Strain CNCM I-4319 Strengthens Gut Functionality

**DOI:** 10.3390/microorganisms8091313

**Published:** 2020-08-28

**Authors:** Rebeca Martín, Francesca Bottacini, Muireann Egan, Celia Chamignon, Valérie Tondereau, Raphaël Moriez, Jan Knol, Philippe Langella, Hélène Eutamene, Tamara Smokvina, Douwe van Sinderen

**Affiliations:** 1 Commensal and Probiotics-Host Interactions Laboratory, Micalis Institute, INRA, AgroParisTech, Université Paris-Saclay, 91190 Jouy-en-Josas, France; rebeca.martin-rosique@inrae.fr (R.M.); celia.chamignon@inrae.fr (C.C.); philippe.langella@inrae.fr (P.L.); 2 APC Microbiome Ireland, University College Cork, T12 K8AF Cork, Ireland; f.bottacini@umail.ucc.ie (F.B.); muireann.egan@umail.ucc.ie (M.E.); 3 Neurogastroenterology & Nutrition Group, Toxalim INRAE, Université de Toulouse, ENVT, INP-Purpan, 31058 Toulouse, France; valerie.tondereau@inrae.fr (V.T.); helene.eutamene@inra.fr (H.E.); 4 Danone Nutricia Research, 91767 Palaiseau, France; raphael.moriez@danone.com; 5 Danone Nutricia Research, 3584 CT Utrecht, The Netherlands; jan.knol@danone.com; 6 Laboratory of Microbiology, Wageningen University, 6708 PB Wageningen, The Netherlands; 7 School of Microbiology, University College Cork, T12 K8AF Cork, Ireland

**Keywords:** bifidobacteria, gut microbiota, gut commensal, gastrointestinal tract, gut health

## Abstract

Bifidobacteria are among the first colonisers of the gastrointestinal tract of breast-fed newborns due to, among other things, their ability to metabolise oligosaccharides naturally occurring in human milk. The presence of bifidobacteria in the infant gut has been shown to promote intestinal health and homeostasis as well as to preserve a functional gut barrier, thus positively influencing host health and well-being. Among human-associated gut commensals, *Bifidobacterium bifidum* has been described as the only species capable of the extracellular degradation of both mucin-type glycans and HMOs, thereby giving this species a special role as a commensal gut forager of both host and diet-derived glycans. In the present study, we assess the possible beneficial properties and probiotic potential of *B. bifidum* strain CNCM I-4319. In silico genome analysis and growth experiments confirmed the expected ability of this strain to consume HMOs and mucin. By employing various animal models, we were also able to assess the ability of *B. bifidum* CNCM I-4319 to preserve gut integrity and functionality from stress-induced and inflammatory damage, thereby enforcing its potential as an effective probiotic strain.

## 1. Introduction

Bifidobacteria represent a group of commensal microorganisms that commonly inhabit the gastrointestinal (GI) tract of various animals, especially humans and other mammals [1]. They represent the most abundant taxon of the infant gut microbiota (their relative abundance can reach 90%), with certain species being particularly prevalent and abundant among the gut microbiota of vaginally-delivered, breast-fed infants, where their high numerical presence is believed to reflect a positive health status [2,3]. As ubiquitous inhabitants of the human gut, these bacteria are purported to play an important role in promoting human health by providing protection against pathogens, stimulating the development and maturation of the immune system during infancy, as well as reinforcing the intestinal barrier throughout human life [4].

Bifidobacteria are saccharoclastic microorganisms, which means that their successful gut colonisation relies to a large degree on their ability to utilise a variety of dietary and host-derived glycans, such as human milk oligosaccharides (HMOs) and mucin-type *O*-glycans [5,6]. The carbohydrate-focused metabolism of bifidobacteria is reflected by their genome, which typically encodes a range of extracellular and intracellular glycosyl hydrolases (GHs), allowing the degradation, internalisation and metabolism of a variety of saccharidic compounds [5,7,8,9]. Comparative genome analyses have provided insightful information concerning the metabolic capabilities of bifidobacteria [10,11,12,13,14]. In fact, the *Bifidobacterium* pan-genome consists of one of the largest predicted glycobiomes among gut commensals, encoding a large number of predicted carbohydrate-active enzymes, including GHs, glycosyl transferases (GTs), and carbohydrate esterases [6].

Several bifidobacterial species, especially those more prevalent in early life (e.g., *Bifidobacterium breve*, *Bifidobacterium bifidum*, *Bifidobacterium longum* and *Bifidobacterium kashiwanohense*) possess metabolic abilities to utilise (certain) HMOs, which represent the third most abundant solid component of human milk [15,16,17,18,19]. Interestingly, this HMO-utilising metabolic property provides those species with a competitive advantage to allow colonisation of and persistence in the (exclusively) breast-fed infant gut [20].

In contrast, only one human-associated bifidobacterial species, *B. bifidum*, has been shown to be a direct utiliser of intestinal mucin-type *O*-glycans, which represent host-derived growth substrates originating from the gut mucus layer surrounding the intestinal epithelium [21,22,23]. Notably, a complete mucin degradation pathway has been characterised in this species [11,15]. Members of *B. bifidum* are also specialised in the degradation and utilisation of host-derived HMOs [7,15,17,24] and possess several extracellular enzymes, allowing this species to serve as a primary degrader of such complex glycans. These extracellular enzymes release free monosaccharides which may subsequently be used by other (bifido)bacteria, thereby allowing cross-feeding and glycan foraging behaviour in the gut [25,26,27,28,29]. For this reason, *B. bifidum* is considered a key player in the establishment of the infant gut microbiota, contributing to the co-evolution between host and its associated bacterial gut community [30].

Maintenance of a functional gut barrier and preservation of intestinal homeostasis are crucial activities underpinning neonatal development. The intestinal mucosal barrier constitutes an effective defence mechanism regulated by both endogenous and exogenous factors involving the integrity of the intestinal epithelial cells and their associated junctions [31,32,33]. When this barrier function is compromised, the consequential higher local antigen exposure is likely to activate the host immune system, thereby causing intestinal inflammation [34,35]. Low-grade gut inflammation has been associated with several diseases and syndromes, such as infantile colic, which is one of the early manifestations of functional GI disorders [36]. Furthermore, a disturbed gut barrier function is often correlated with visceral hypersensitivity, a phenomenon that may, in turn, catalyse the onset of functional gastrointestinal diseases [37]. Beneficial gut commensals such as particular strains of lactic acid bacteria (LAB) and bifidobacteria have been shown to protect and repair the gut barrier [38,39], where such protection and/or restoration may occur through a variety of mechanisms [35,40,41,42,43,44].

The objective of the current study was to assess possible beneficial properties of *B. bifidum* strain CNCM I-4319 that may be relevant for healthy gut development in newborns. Genome analysis and growth experiments were undertaken to study the carbohydrate metabolism of this strain, with particular focus on the consumption of HMOs and mucin. Moreover, the ability of *B. bifidum* CNCM I-4319 to interact with its host and protect the host intestine from stress-induced and inflammatory damage was assessed using two animal models.

## 2. Materials and Methods

### 2.1. Genome Sequencing, Assembly, and Bioinformatic Analyses

Genome sequencing of *B. bifidum* CNCM I-4319 was performed using the Pacific Bioscience SMRT RSII sequencing platform (PacBio, Menlo Park, CA, USA). The obtained raw reads were assembled with the Hierarchical Genome Assembly Process (HGAP) pipeline using the protocol RS_Assembly.2 implemented in SMRT Smart Analysis portal v.2.3 (https://www.pacb.com/support/software-downloads/). Automatic annotation of predicted open reading frames (ORFs) was performed using a combination of PRODIGAL v.2.6.3 (https://github.com/hyattpd/Prodigal) and BLASTP alignments [45] to assign annotation (using an E-value cut-off of 0.0001 for hits showing at least 50% of similarity across at least 50% of the sequence length) against a non-redundant protein database provided by the National Centre for Biotechnology Information portal (http://www.ncbi.nlm.nih.gov/). Where appropriate, automatic annotation was refined with information obtained from similarity searches involving alternative databases such as protein family (Pfam) [46] and COG [47]. The generated GenBank file was further inspected in Artemis v.16 [48] and, where necessary, manual redefinition of the start codons or removal/addition of predicted coding regions were performed. Ribosomal RNA (rRNA) and transfer RNA (tRNA) genes were detected using RNAMMER v1.2 (http://www.cbs.dtu.dk/services/RNAmmer/) and tRNA-scanSE v2.0 (http://lowelab.ucsc.edu/tRNAscan-SE/), respectively. The presence of Restriction/Modification systems was determined through interrogation of the REBASE database (http://rebase.neb.com/rebase/rebase.html), as described previously [49].

Following assembly and annotation, the genome sequence underwent methylome analysis where modified bases were detected using the “RS_Modification_and_Motif_Analysis.1” protocol implemented in the SMRT Analysis Portal v.2.3 (https://www.pacb.com/support/software-downloads/). Only methylation motifs showing a score higher than 40 (corresponding to a *p* value of <0.0001) were considered significant (https://github.com/PacificBiosciences/Bioinformatics-Training/wiki/Methylome-Analysis-Technical-Note). Complete and draft *B. bifidum* genomes were retrieved from the Genbank ftp repository (ftp://ftp.ncbi.nih.gov/genbank/) available from the National Centre for Biotechnology Information (http://www.ncbi.nlm.nih.gov/). Comparative analysis was performed using a combination of all-against-all, bi-directional BLASTP alignments [45] (cut-off: E-value < 0.0001, with at least 50% identity across at least 50% of either protein sequence), and the Markov Cluster Algorithm (MCL) implemented in the mclblastline pipeline v12-0678 [50]. The resulting gene families were classified as belonging to the so-called core or variable genome based on their presence in either all strains or in a subset of the investigated strains, respectively, as described previously [51].

### 2.2. Optimal Growth Conditions and Carbohydrate Utilisation Abilities of B. bifidum CNCM I-4319

*B. bifidum* CNCM I-4319 was routinely cultured in modified MRS (mMRS) prepared from first principles [51], and supplemented with 0.05% (wt/vol) cysteine-HCl and 0.5% (wt/vol) of a given carbohydrate before inoculation. Bacterial cultures were incubated anaerobically for 24 h at 37 °C, and optical density (OD_600nm_) values were determined at various time points to assess growth.

To analyse carbohydrate fermentation abilities of strain CNCM I-4319, 10% stock solutions of each carbohydrate were first prepared in distilled water, followed by filter sterilisation. For carbohydrates with lower solubility (i.e., arabinoxylan, xylan, arabinan, arabinogalactan, galactan, starch, pullulan and mucin), a 0.5% solution in mMRS was prepared and sterilised by autoclaving. In order to determine bacterial growth profiles, 10 mL of a freshly prepared mMRS medium, supplemented with 0.05% (wt/vol) cysteine-HCl and 0.5% (wt/vol) of the carbohydrate of interest, was inoculated with 100 µL (1%) of an overnight (i.e., stationary phase) culture of a particular strain. Cultures were incubated anaerobically for 24 h, and the OD_600nm_ was determined at regular intervals. mMRS (including cysteine-HCl) without the addition of a carbohydrate source was used as a negative control.

### 2.3. Low-Grade Inflammation (LGI) Model

Induction of low-grade inflammation in C57BL/6 mice (Janvier, Le Genest Saint Isle, France) by intrarectal administration of dinitrobenzene sulfonic acid (DNBS) solution (ICN, Biomedical Inc., Clinton Township, MI, USA) and measures of inflammatory parameters (weight loss and myeloperoxidase activity) were performed as previously described [44,52]. Mice received 5 × 10^9^ CFU/mL of viable *B. bifidum* CNCM I-4319 in 200 µL of PBS or PBS alone intra-gastrically through daily administrations for 10 days (Figure S1, panel A). Animal care and work protocols were approved by the local regional ethical committee (Comethea) according to EU directive 2010/63/EU.

### 2.4. Water Avoidance Stress (WAS) Model and Visceral Sensitivity Measures

Chronic water avoidance stress (WAS) on male and female Wistar rats (220–250 g; Janvier SA, Le Genest St Isle, France) was delivered according to a slightly modified procedure [53] for 1 h for 9 days between 8 and 10 A.M. For the non-WAS group (control group), a similar procedure was performed with an empty plastic container. Animals received *B. bifidum* CNCM I-4319 (total cell count 9.2 × 10^9^ CFU/mL, final gavage volume 1 mL), or a 0.9% NaCl (saline) solution for the control group, by gavage for 15 days (Figure S1, panel B). To evaluate visceral sensitivity, abdominal contractions were measured in female rats as previously described [54]. Animal care and work protocols were approved by the local regional ethical committee (Midi-Pyrenees) according to the EU directive 2010/63/EU.

### 2.5. Intestinal Permeability Determinations in WAS and LGI Models

In the WAS model, gut permeability was evaluated in male rats using ^51^Cr ethylene diamine tetra acetic acid (^51^Cr-EDTA; Perkin Elmer Life Sciences, Paris, France) as a marker of paracellular permeation of tight junctions [55]. At the end of the treatment period (i.e., day 15), following the final WAS or non-WAS session, animals received an oral administration of ^51^Cr-EDTA. ^51^Cr-EDTA (25.9 kBq) was diluted in 0.5 mL of saline and administered by gavage. Rats were then placed in metabolic cages and radioactivity in urine was measured with a gamma counter (Cobra II; Packard). Permeability to ^51^Cr-EDTA was expressed as a percentage of total radioactivity administered.

In the LGI model, gut permeability was determined using fluorescein-conjugated dextran (FITC-dextran 3000–5000 Da (FD-4), Sigma-Aldrich, St. Louis, MO, USA) tracer at the endpoint [52]. Briefly, 0.6 mg/g body weight of FD-4 dissolved in PBS was administered to mice by oral gavage and 3.5 h after blood samples were recovered from the retro-orbital venous plexus and kept in the dark at 4 °C until analysis. Serum was separated by centrifugation and plasma FITC levels were determined using a fluorescence microplate reader (excitation 485 nm, emission 530 nm, Tecan, Lyon, France).

### 2.6. Histological Analysis

Flushed colons were fixed in Carnoy buffer, dehydrated and embedded in paraffin following a standard protocol. Tissue blocks were cut into thin sections (5 µm) on a Leica RM2265 microtome and mounted on adhesive microscope slides (Superfrost ultra plus, ThermoScientific, Waltham, MA, USA). Histological features were analysed by Alcian blue (AB) staining [56,57]. For mucin-2 (Muc2) detection, samples were isolated (Dako Pen, Agilent Technologies, Santa Clara, CA, USA) and incubated sequentially with: the Protein block (Dako, Agilent Technologies), the primary antibody (2 μg/mL, Mucin 2 rabbit polyclonal IgG, Santa Cruz Biotechnologies, Dallas, TX, USA) and the secondary antibody (2 ng/mL, Alexafluor 568 goat red anti-rabbit IgG, Invitrogen, ThermoFischer Scientific, Waltham, MA, USA) both diluted in Ab diluent (Dako, Agilent Technologies). Sections were then treated with trihydro-chloride trihydrate (0.5 mg/mL Hoechst 33342, Invitrogen, ThermoFischer Scientific) in PBS. The slides were mounted using fluorescent mounting medium (Dako, Agilent Technologies). Tissues were visualized using a high capacity digital slide scanner (3DHISTECH Ltd., Budapest, Hungary) and the Panoramic and Case viewer software (3DHISTECH Ltd.)

### 2.7. Analyses of Lymphoid Population

Mononuclear cells were isolated from spleens and mesenteric lymphoid nodes (MLN) by gentle extrusion of the tissue through a 50 µm-mesh Nylon cell strainer (BD Biosciences, San Jose, CA, USA) and subsequent lysis of erythrocytes in red blood cell lysing buffer (Sigma-Aldrich) as previously described [52].

For flow cytometry analysis, aliquots of 10^6^–10^7^ cells per sample were pre-incubated with purified anti-mouse CD16/CD32 (eBioscience, San Diego, CA, USA) and then labelled with anti-CD3-FITC, anti-CD4-PerCP, anti-Tbet-APC and anti-Gata3-PE (all from eBioscience) according to the manufacturer’s instructions. Intracellular staining was performed with the FoxP3 Staining kit (eBioscience). Stained cells were analysed by flow cytometry (Accuri, Ann Arbor, MI, USA) with CFlowSampler software (BD Accuri, Ann Arbor, MI, USA).

For stimulation experiments, 2 × 10^5^ cells isolated from spleens or MLN were cultured for 48 h (37 °C, 10% CO_2_) in DMEM medium in P24 plates pre-coated with anti-CD3/CD28 antibodies (4 µg/mL each; eBioscience) or phorbol 12-myristate 13-acetate (PMA)/ionomycin (cell stimulation cocktail, 1×, ebioscience). Culture supernatant was frozen at −80 °C until processing and cytokine concentration determined by a cytometric bead array system (Mouse Th1/Th2/Th17/Th22 13plex Flowcytomix) (eBioscience) according to the manufacturer’s instructions.

### 2.8. Statistical Analysis

GraphPad software (GraphPadSofware, La Jolla, CA, USA) was used for statistical analysis. Results were presented as dot plots with median +/− interquartile range. Comparisons involved the non-parametric Kruskal–Wallis test followed by a Dunn’s multiple comparison test. A *p* value of less than 0.05 was considered significant. The results of visceral pain response were analysed by one-way ANOVA followed by Tukey’s post hoc test.

## 3. Results and Discussion

### 3.1. General Features of B. bifidum CNCM I-4319

*B. bifidum* CNCM I-4319 is a strain originally isolated from the infant microbiota of a healthy baby born in The Netherlands. This strain was selected due to its ability to protect the intestinal epithelial barrier measured by transepithelial electrical resistance (TEER) in TNF-α-induced hyperpermeability Caco2 model (unpublished data).

**Figure 1:**
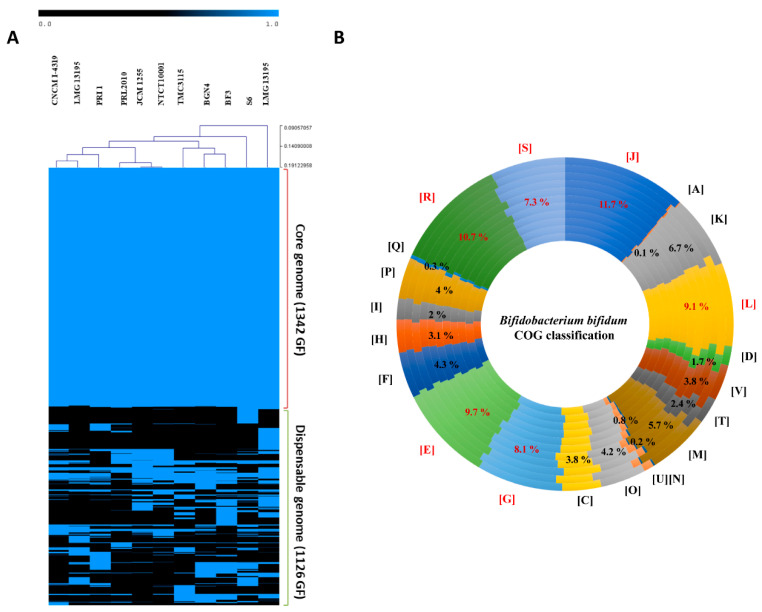
Comparative genomics of *B. bifidum* CNCM I-4319. (**A**) Hierarchical clustering heatmap representing the variability of *B. bifidum* in terms of presence/absence of gene families computed using all-vs.-all blastp alignment and MCL clustering. The number of core (present in all genomes) and dispensable (present in some genomes) gene families are also indicated. (**B**) Cluster of Orthologues (COG) classification of the *B. bifidum* families of orthologues. For each COG entry, the average percentage of hits among the species is indicated. COG classification: [D] cell cycle control, cell division, chromosome partitioning; [M] cell wall/membrane/envelope biogenesis; [N] cell motility; [O] post-translational modification, protein turnover, and chaperones; [T] signal transduction mechanisms; [U] intracellular trafficking, secretion, and vesicular transport; [V] defence mechanisms; [A] RNA processing and modification; [J] translation, ribosomal structure and biogenesis; [K] transcription; [L] Replication, recombination and repair; [C] energy production and conversion; [E] amino acid transport and metabolism; [F] nucleotide transport and metabolism; [G] carbohydrate transport and metabolism; [H] Coenzyme transport and metabolism; [I] lipid transport and metabolism; [P] inorganic ion transport and metabolism; [Q] secondary metabolites biosynthesis, transport, and catabolism; [R] general function prediction only; [S] function unknown. The most abundant families are highlighted in red and they are assigned to housekeeping functions.

Genome sequencing of *B. bifidum* CNCM I-4319 resulted in a single contig representing a chromosome of 2,193,720 base pairs (bp), with a G + C% content of 62.70%, in line with other publicly available bifidobacterial genomes [12,58]. The genome of *B. bifidum* CNCM I-4319 was predicted to harbour 1759 genes, 3 rRNA loci and 53 tRNAs, values that are in line with previous reports [12,58] (Table 1). According to the Cluster of Orthologous Groups (COG) classification, the highest percentage of predicted proteins in this strain is involved in translation, ribosomal structure and biogenesis (12%), followed by amino acid transport and metabolism (10%) and carbohydrate transport and metabolism (9%) (Figure 1, panel B), values that are similar to those previously reported [12].

Comparative analysis was performed in order to compare *B. bifidum* CNCM I-4319 with 10 other fully sequenced *B. bifidum* genomes (Table 1). BLASTP alignment and MCL clustering analysis identified the presence of a total of 2468 gene families contained in the 11 *B. bifidum* genomes analysed here. Of these, 1342 are present among all strains (thus constituting the core-genome), while the remaining 1126 are classified as accessory gene functions as they are only present in one or a sub-groups of the assessed strains (Figure 1, panels A,B). Whole-genome alignment employing *B. bifidum* CNCM I-4319 as a reference strain against other *B. bifidum* genomes showed that the obtained genome sequence of CNCM I-4319 is highly syntenic with other members of this species, with the exception of *B. bifidum* TMC3115, which contains an apparent genomic inversion (Figure S2).

**Figure 2:**
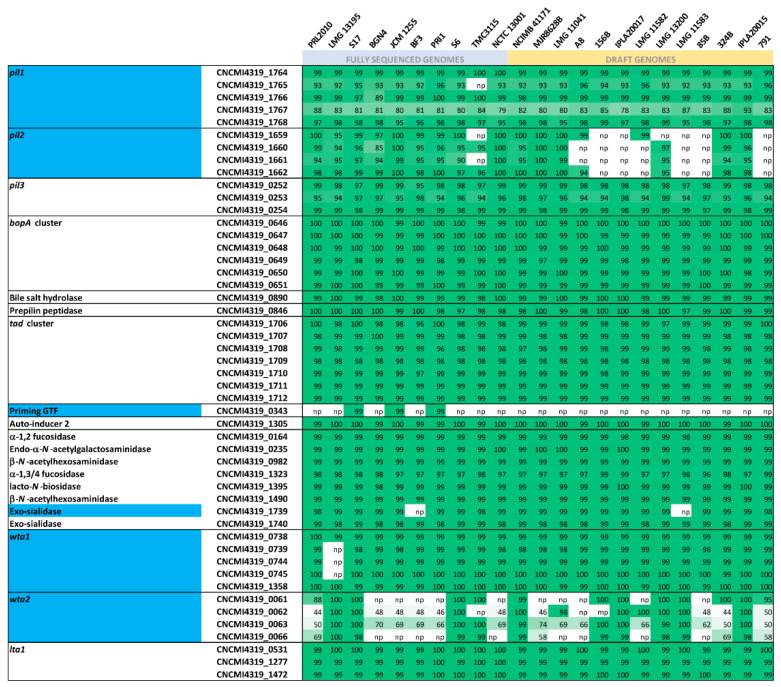
Adhesion and host-interacting features of *B. bifidum* CNCM I-4319. Heatmap showing the distribution of *B. bifidum* CNCM I-4319 gene clusters which are predicted to encode probiotic properties and/or responsible for host-interaction and gut colonisation. The green scale indicates sequence similarity (BLASTP alignment) of each identified CNCM I-4319 gene with its homologous in sequenced *B. bifidum* strains. Highlighted in blue represent clusters that are found to be variably distributed across members of the *B. bifidum* species.

Notably, the in silico search for extracellular and host-interacting genomic features of *B. bifidum* CNCM I-4319 combined with comparative genome analysis identified 49 key genes which were identified as part of the core or dispensable genome of this species (Figure 2). For example, a typical bifidobacterial Tad pilus gene cluster homologous to the one first characterised in *B. breve* UCC2003 and essential for gut colonisation and persistence [59,60] was found in the genome of *B. bifidum* CNCM I-4319 (corresponding to locus tags CNCMI4319_1706–1712). Comparative analysis showed that this locus is also fully conserved among *B. bifidum* representatives (Figure 2). In contrast, among the three identified sortase-dependent pilus loci (here named *pil1, pil2* and *pil3*), the *pil2* locus (corresponding to locus tags CNCMI_1659–1662) appears to be variably present among the assessed representatives of this species (Figure 2). Notably, this cluster also shows high homology (between 94 and 100% identity) with the *pil2* cluster first described in *B. bifidum* PRL2010, which is expressed in vivo and is responsible for adhesion to a Caco-2 human intestinal epithelial cell line [61].

BopA is a lipoprotein that is abundantly expressed on the cell surface of *B. bifidum* [62] and was previously shown to adhere to Caco-2 epithelial cells [63,64]. The *bopA* gene cluster identified in *B. bifidum* CNCM I-431 (corresponding to locus tags CNCMI4319_0646–0651) is also highly conserved in the *B. bifidum* species which is in accordance with a previous report [63].

Members of the *B. bifidum* species are proficient at utilising host-derived carbohydrates such as mucin as a sole carbon source [15]. Genes that are predicted to encode extracellular enzymes required to fully degrade mucin, including two putative exo-α-sialidases (CNCMI4319_1739-40), two putative α-1,2/3 fucosidases (CNCMI4319_0164 and CNCMI4319_1323), an endo-α-*N*-acetylgalactosaminidase (CNCMI4319_0235) and *N*-acetyl-β-hexosaminidases (CNCMI4319_0982 and CNCMI4319_1490) were all identified in the *B. bifidum* CNCM I-4319 genome (Figure 2). Comparative genome analysis revealed that the genes encoding these enzymes are typically conserved across the species, in agreement with previous reports and the fact that mucin degradation constitutes a key feature of *B. bifidum* [11,15].

### 3.2. B. bifidum CNCM I-4319 Methylome Analysis

Methylome analysis of *B. bifidum* CNCM I-4319 identified seven methylated motifs, all of which are methylated at an adenine base (Table 2). Motifs 1 and 2, 3 and 4, and 5 and 6 represent complementary, asymmetric recognition sequences, typical of type I restriction-modification (R/M) systems. Motif 7 shares sequence similarity with motif 6 and is therefore assumed to be a sub-motif recognised by the same R/M system. The annotated genome of *B. bifidum* CNCM I-4319 was also compared against the REBASE database. Three potential type I R/M systems were identified, corresponding to locus tags CNCMI4319_0147–0151, CNCMI4319_1112–1117 and CNCMI4319_1327–1330, which is in agreement with the methylome analysis data. These predicted type I systems include a methyltransferase, a restriction endonuclease and one or more specificity subunits, characteristic of this type of R/M system [65].

In addition, a type III R/M system was predicted to be encoded by this genome (corresponding to locus tags CNCMI4319_0898–0891), although apparent frameshift mutations in both the methyltransferase and the restriction endonuclease-encoding genes likely render this system non-functional. Furthermore, three separate type I methyltransferases at locus tags CNCMI4319_0284, CNCMI4319_0408 and CNCMI4319_0825 were identified using REBASE. These genes share high sequence similarity with other methyltransferases in the REBASE databases. However, their accompanying restriction endonucleases could not be identified. Notably, the methyltransferases encoded by the genes at locus tags CNCMI4319_0408 and CNCMI4319_0825 are likely to be pseudogenes because of their small size, thus representing non-functional orphan methyltransferases.

### 3.3. Carbohydrate Utilisation Profiles of B. bifidum CNCM I-4319

Carbohydrate utilisation abilities of *B. bifidum* CNCM I-4319 were tested on 46 different carbohydrates, the results of which are summarised in Table 3.

*B. bifidum* CNCM I-4319 was shown to exhibit growth on 16 of the 46 tested sugars, predominantly host-derived sugars such as human milk oligosaccharides (HMO) and mucin. No or weak growth was observed on the monosaccharide components of mucin and HMO, such as fucose, sialic acid, galactose, *N*-acetylglucosamine; however, the disaccharide lactose was shown to support good growth. This is a characteristic of other members of the *B. bifidum* species, which typically encode a number of extracellular glycosyl hydrolases, the purpose of which is to cleave large, complex carbohydrates such as mucin-associated *O*-glycans and HMOs. *B. bifidum* strains are known to be able to metabolize constituents of these complex glycans, such as lactose, *N*-acetyllactosamine, LNB and galacto-*N*-biose (GNB) [66]. Consistent with this, we identified genes encoding two sialidases, fucosidases, β-*N*-acetylhexosaminidases, a lacto-*N*-biosidase and an endo-α-*N*-acetylgalactosaminidase, all predicted to be extracellular, as well as a putative LNB/GNB utilisation cluster on the genome of *B. bifidum* CNCM I-4319.

### 3.4. B. bifidum CNCM I-4319 Inhibits Visceral Sensitivity Due to Colorectal Distension in Rats

**Figure 3:**
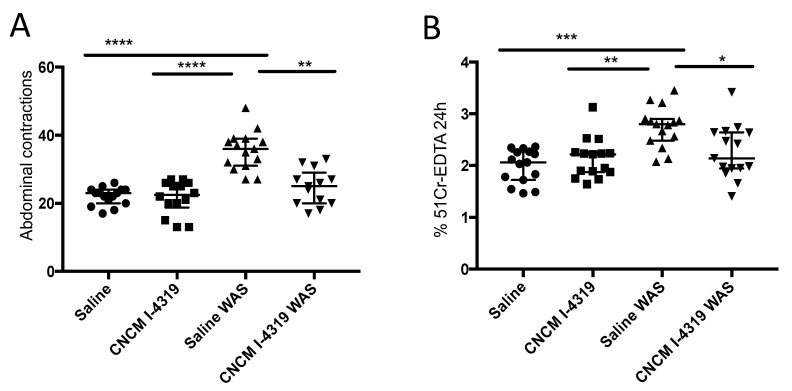
Visceral sensitivity and permeability measures in WAS model. Abdominal contractions in response to colorectal distension (**A**) and in vivo permeability measured by 51Cr-EDTA (**B**). Control no stressed (saline), control stressed (saline WAS), group no stressed treated with *B. bifidum* CNCM I-4319 strain (CNCM-I4319), group stressed treated with *B. bifidum* CNCM I-4319 strain (CNCM-I4319 WAS). *: *p* < 0.05; **: *p* < 0.01; ***: *p* < 0.001, ****: *p* < 0.0001.

In order to evaluate the impact of *B. bifidum* CNCM I-4319 on the level of visceral sensitivity in WAS-treated rats, we measured the frequency of abdominal contractions. WAS is a chronic stress model which affects visceral sensitivity to pain and which can be modulated through alterations in the gut microbiota [67]. As expected, colorectal distension (CRD) increased the frequency of abdominal contractions per 5 min in a volume-dependent manner. In rats treated with saline in basal condition, the first volume of distension that significantly (*p* < 0.01) increased the number of abdominal contractions was 0.8 mL. Therefore, we used 0.8 mL as the reference volume in this study, representing a visceral hypersensitive response. Under control conditions (i.e., non-WAS exposed session), *B. bifidum* CNCM I-4319 did not modify the visceral sensitivity response to CRD (Figure 3, panel A). In saline-treated animals, WAS significantly increased the number of abdominal contractions when compared to the control (22.07 ± 0.67 vs. 35.47 ± 1.46; *p* < 0.05) (Figure 3, panel A). Chronic oral treatment with *B. bifidum* CNCM I-4319 showed a clear and significant reduction in the number of abdominal contractions induced by WAS (35.47 ± 1.46 vs. 24.69 ± 1.47; *p* < 0.05).

These findings support the notion that CNCM I-4319 is able to inhibit stress-induced visceral hyperalgesia in rats.

### 3.5. B. bifidum CNCM I-4319 Prevents the Increase in Gut Permeability in Rats

In humans, gut hypersensitivity has been linked to an increase in intestinal permeability, where epithelial intestinal permeability impairment is positively correlated with visceral pain development [68]. In this context, probiotics have been reported to play a protective role in stress-induced alterations of gut functions by modulating the intestinal barrier via a reduction in paracellular gut permeability [67]. In our study, in animals treated with saline (saline group), the imposition of WAS significantly (*p* < 0.05) increased colonic paracellular permeability to ^51^Cr-EDTA in comparison with control conditions (i.e., animals not subjected to a WAS session) (2.75 ± 0.10% vs. 1.98 ± 0.08%). Under control conditions (i.e., no WAS-imposed stress), orally and recurrently administered *B. bifidum* CNCM I-4319 had no significant impact on gut paracellular permeability (1.98 ± 0.08% vs. 2.14 ± 0.10%). Regularly administered *B. bifidum* CNCM I-4319 was shown to prevent the increase in intestinal paracellular permeability induced by WAS in a significant manner (2.75 ± 0.10% vs. 2.26 ± 0.13%; *p* < 0.05) (Figure 3, panel B), with values corresponding to those observed under control conditions (i.e., in the absence of WAS-imposed stress).

### 3.6. B. bifidum CNCM I-4319 Alleviates Moderate Inflammatory Symptoms Induced by a Chronic Low Dose DNBS Challenge in Mice

**Figure 4:**
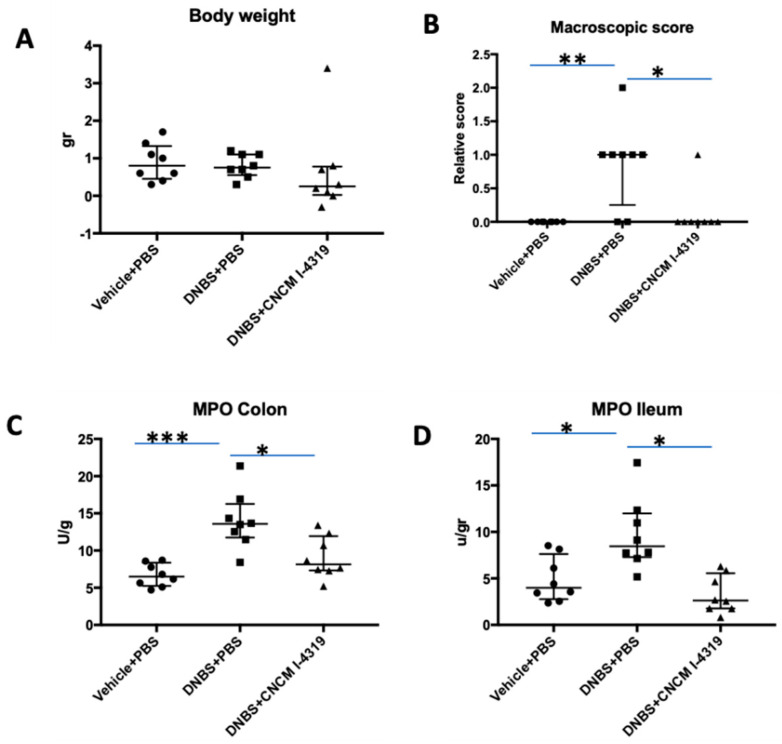
General health parameters in LGI model. Weight loss (**A**), macroscopic score (**B**), and colon and ileum MPO activity (**C**,**D**). Control non-inflamed (vehicle-PBS), control inflamed (dinitrobenzene sulfonic acid (DNBS)-PBS), *B. bifidum* CNCM I-4319 strain (DNBS-CNCM-I4319). *: *p* < 0.05; **: *p* < 0.01; ***: *p* < 0.001.

Low-grade inflammation is a condition closely related to several diseases and gastrointestinal syndromes, such as irritable bowel disease (IBS), as well to gut comfort and barrier impairment. In our study, mice were subjected to a low-grade chronic inflammation-inducing protocol in order to mimic the gut barrier perturbations induced by this microinflammation. The presence of a modest yet measurable inflammation status was validated through the absence of significant body weight changes during the recovery period (Figure 4, panel A), and slight increases in macroscopic score and colonic and ileac myeloperoxidase (MPO) values (Figure 4, panels B–D). Notably, *B. bifidum* was shown to be able to alleviate these minor inflammatory effects in a significant way (*p* < 0.05) (Figure 4).

It is worth noting that the immature gut of newborns is characterised by a higher level of permeability, which is necessary to increase exposure to microbial and food antigens during the maturation of the immune system [69]. Therefore, our results indicate that *B. bifidum* is able to alleviate the effects of low-grade inflammation that occur during the developmental stage of the gut.

### 3.7. B. bifidum CNCM I-4319 Restores Colonic Permeability and Goblet Cell Populations Altered by DNBS Chronic Challenge and Increases Mucus Production in Mice

**Figure 5:**
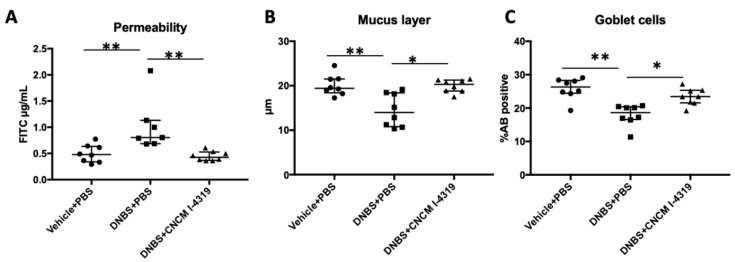
In vivo permeability measurements and effect on Goblet cells and mucus production in LGI model. For in vivo measurements of gut permeability, animals were orally gavaged with FITC-dextran (**A**). Percentage of positive cells stained with AB (Alcian Blue) and mucus layer thickness measured by muc-2 immunohistochemistry (**B**,**C**). Control non-inflamed (vehicle-PBS), control inflamed (DNBS-PBS), *B. bifidum* CNCM I-4319 strain (DNBS-CNCM-I4319). *: *p* < 0.05 **: *p* < 0.01.

The DNBS-induced LGI model is known to perturb gut barrier structure and integrity, which affects gut functionality [70]. In order to assess the remedial effects elicited by *B. bifidum* CNCM I-4319 on gut barrier integrity, the in vivo permeability was analysed by measuring the diffusion level of the paracellular tracer FITC-dextran. Administration of *B. bifidum* was shown to counter the increase in permeability induced by the imposed chronic DNBS challenge (*p* < 0.05) (Figure 5, panel A). Furthermore, treatment with this bacterium was shown to enhance the percentage of goblet cells, while the thickness of the mucus layer (Figure 5, panels B and C) was also shown to be positively affected in this chronic gut dysfunction model in a significant way (*p* < 0.05). The gut barrier is a complex biological unit organised as a multilayer tissue that is composed of (i) a physical barrier which prevents bacterial adhesion and regulates paracellular diffusion, and (ii) a functional layer able to discriminate between beneficial and undesired microorganisms [71]. The physical barrier is formed of a mucus layer, followed by a layer of epithelial cells [35,72]. The mucus layer protects the epithelium from external attacks (harmful microorganisms or antigens), while also being a lubricant mediating intestinal motility [71]. It is composed of two layers—a compact inner layer and a looser outer one. Both layers are mainly composed of mucin MUC2, which is produced by goblet cells [71]. In fact, mucus production has previously been shown to be affected during inflammation with intestinal dysbiosis [73], and several bifidobacterial strains have been found to be able to induce mucus production and/or adhere to it [74,75].

Our results indicate that *B. bifidum* has beneficial effects on permeability and low-grade inflammation parameters, which may, at least in part, be due to its protective role at the structural gut barrier level by increasing mucus production and goblet cell populations (Figure 5). Indeed, certain bifidobacterial strains have previously been reported to increase mucus production through their ability to produce acetate, which is a major metabolite of their fermentative lifestyle [74,75]. The fact that strain CNCM I-4319, like other *B. bifidum* strains, is able to use mucin as a fermentable substrate (see above) suggests that this strain resides in close proximity to the mucus layer where its metabolism causes localised acetate production with a consequent increase in mucin biosynthesis.

### 3.8. B. bifidum CNCM I-4319 Modulates CD3+/CD4+T-Cell Populations in Spleen and Mesenteric Lymphoid Nodes (MLN)

**Figure 6:**
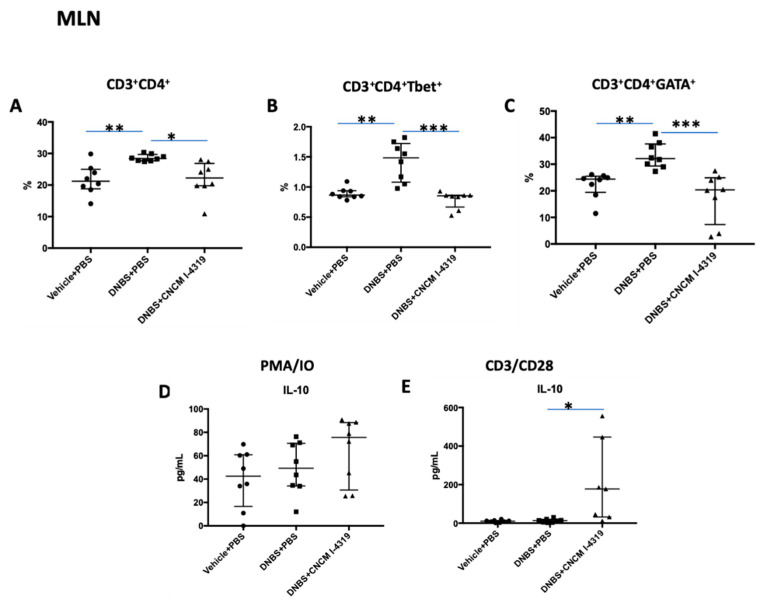
MLN population levels in LGI model. CD3/CD4-positive cells and subsets detected by flow cytometry (**A**–**C**) and cytokine production in MLN cultures stimulated with CD3^+^/CD28^+^ or PMA/IO (**D**,**E**). Control non-inflamed (vehicle-PBS), control inflamed (DNBS-PBS), *B. bifidum* CNCM I-4319 strain (DNBS-CNCM-I4319). * *p* < 0.05 ** *p* < 0.01 *** *p* < 0.001.

**Figure 7:**
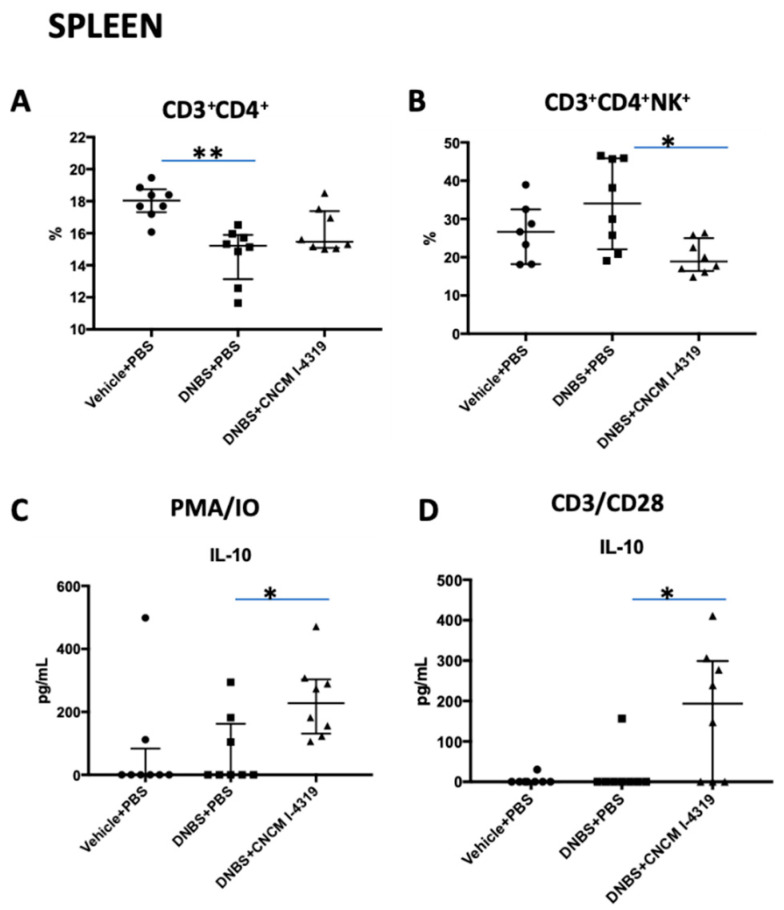
Splenocyte population levels in LGI model. CD3/CD4-positive cells and subsets detected by flow cytometry (**A**,**B**) and cytokine production in splenocyte cultures stimulated with CD3^+^/CD28^+^ or PMA/IO (**C**,**D**). Control non-inflamed (vehicle-PBS), control inflamed (DNBS-PBS), *B. bifidum* CNCM I-4319 strain (DNBS-CNCM-I4319). *: *p* < 0.05, **: *p* < 0.01.

Changes in mucosal permeability such as those observed in the DNBS chronic low dose model can be the cause or the consequence of a modest immune response. The DNBS-mediated chronic LGI protocol is known to modulate lymphocyte populations in spleen and MLN, and to alter Th1 and Th2 profiles [70]. To investigate possible mechanisms by which *B. bifidum* CNCM I-4319 exerts its protective effects, T-cells from the spleen and MLN were isolated and analysed by flow cytometry (Figure 6 and Figure 7). DNBS-treated mice were shown to contain lower CD3^+^/CD4^+^ T cell percentages in their spleen when compared to the control group, and higher CD3^+^/CD4^+^ cell percentages in MLN (*p* < 0.05) (Figure 6 and Figure 7). Interestingly, treatment with *B. bifidum* was demonstrated to control these levels, reaching values that are reminiscent of the non-inflamed control group (*p* < 0.05). Furthermore, *B. bifidum* was able to restore the levels of Tbet- and GATA-positive T-cells in MLN (Th1 and Th2, respectively) and of NK-positive T-cells in the spleen in a significant manner (*p* < 0.05) (Figure 6 and Figure 7). These results indicate that strain CNCM I-4319 is able to restore the Th1/Th2 balance when the latter is distorted as a result of low-grade inflammation, a phenomenon that may have positive consequences for the health status of mice in this animal model.

As variations in CD4+ T-cell populations were found, MLN and spleen cells were isolated and cultivated over 48 h in the presence of either CD28+/CD3+ (to specifically stimulate lymphocytes) or PMA/IO (to stimulate all the cells present in the organ disaggregate). Representative cytokines of the major profiles were determined in culture supernatants. No differences were found for most of the cytokines tested. However, *B. bifidum* treatment was able to increase IL-10 production in both the spleen and MLN, pointing to an anti-inflammatory role of this bacterium even if Treg populations do not appear to be altered (Figure 6, panels D and E; Figure 7, panels C and D).

It is generally accepted that hapten-mediated colon inflammation protocols (TNBS, DNBS) are associated with a Th1 response [76]. Our study confirms that, even in a gut dysfunction model provoked by low-grade inflammation, the DNBS challenge increases the Th1 response. *B. bifidum* was shown to be able to counterbalance this response, in contrast to what has been observed with other bifidobacterial strains in the same colitis model [52]. Indeed, *Bifidobacterium* strains have been found to modulate T cell populations with different results depending on the strain and the model, confirming that immuno-modulatory effects, as well as general probiotic effects, are often strain-specific [27,77].

## 4. Conclusions

Genome analysis and functional characterisation of *B. bifidum* CNCM I-4319 suggest that this strain represents a suitable candidate for probiotic supplementation targeted at infants. In fact, this strain, originally isolated from the microbiota of a newborn, seems to be specifically adapted to colonising the infant intestine and to actively maintaining its integrity and functionality during gut maturation in early life.

The genome sequence and growth performances of CNCM I-4319 strain highlight the preferences of this strain towards the utilisation of human milk oligosaccharides but also mucin, which is a rare characteristic among members of the gut microbiota, especially beneficial gut commensals such as *B. bifidum*. From a functional point of view, this strain was shown to preserve the integrity of the mucus layer by protecting goblet cells (which are mucus producers) in a low-inflammation animal model. These characteristics are likely to support the ability of this strain to colonise and persist in the early-life human intestine.

Our low-grade inflammation animal model shows that *B. bifidum* strain CNCM I-4319 not only possesses anti-inflammatory capacities but also protects the gut from inflammation-induced hyperpermeability. By employing a rat-based stress model, we showed that *B. bifidum* CNCM I-4319 protects the murine host against gut-hypersensitivity that can be induced by psychological stress (i.e., water avoidance).

Taken together, our results indicate that *B. bifidum* CNCM I-4319 represents a strain that is well adapted to colonise the infant intestine. Its presence actively promotes the preservation of gut functionality during immune development that could have short- and long-term health consequences by preventing the low-grade inflammatory status of the intestine, that may otherwise trigger gut hypersensitivity [78,79].

Of course, one must take into consideration that our findings are based on non-human and in vitro models which aside from the strain’s ability to metabolise HMOs do not necessarily reflect a human situation. Nonetheless, our first results clearly support the notion that this strain is a suitable candidate for future clinical interventions, through which further data will be collected to assess its full potential as a probiotic for early life.

## Figures and Tables

**Table 1:** *B. bifidum* genomes used for comparative analysis.

Genome	ORF Number	Genome Size	GC Content (%)	TUG Number	Accession Number
*B. bifidum* CNCM I-4319	1759	2,193,720	62.70	11	CP058603.1
*B. bifidum* PRL2010	1706	2,214,656	62.70	21	CP001840.1
*B. bifidum* LMG 13195	2106	2,261,666	62.60	94	AP018131.1
*B. bifidum* S17	1715	2,186,882	62.80	25	CP002220.1
*B. bifidum* BGN4	1727	2,223,664	62.60	26	CP001361.1
*B. bifidum* JCM 1255	1723	2,211,039	62.70	9	AP012323.1
*B. bifidum* BF3	1696	2,210,370	62.60	30	CP010412.1
*B. bifidum* PRI1	1718	2,243,572	62.70	32	CP018757.1
*B. bifidum* S6	1771	2,311,342	62.70	79	CP022723.1
*B. bifidum* TMC3115	1612	2,178,894	62.80	38	AP018132.1
*B. bifidum* NCTC 13001	1736	2,211,032	62.70	13	LR134344.1

**Table 2:** Methylated motifs of *B. bifidum* CNCM I-4319.

Type	Motif	Modified Position	Methylation
I	GGCANNNNNCTC	4	m6A
I	GAGANNNNNTGCC	2	m6A
I	CAAYNNNNNCTG	3	m6A
I	CAGNNNNNRTTG	2	m6A
I	CGYANNNNNNNTCC	4	m6A
I	GGANNNNNNNTRCG	3	m6A
I	GGANNNNNNNTCC	3	m6A

**Table 3:** Carbohydrate utilisation profile of *B. bifidum* CNCM I-4319.

Carbohydrate Source	Level of Growth
mMRS: no sugar	−
Glucose	+++
Galactose	+
Arabinose	−
Mannose	−
Xylose	−
Ribose	−
Fructose	+++
Rhamnose	−
Fucose	−
*N*-acetylglucosamine	+
Sialic acid	−
Glucuronic acid	−
Galacturonic acid	−
Sorbitol	−
Mannitol	−
Arabitol	−
Xylitol	−
Lactose	+++
Lactulose	+++
Maltose	+
Isomaltulose	−
Sucrose	−
Cellobiose	−
Turanose	−
Melibiose	+
Raffinose	−
Melezitose	−
6′ sialyllactose	+++
2′-fucosyllactose	+++
3-fucosyllactose	+++
Lacto-*N*-tetraose	+++
Lacto-*N*-neotetraose	+++
Lactosamine-HCl	+++
Xylo-oligosaccharides	−
Arabinoxylan	−
Xylan	−
Arabinan	−
Arabinogalactan	−
Galactan	−
Starch	−
Pullulan	−
Mucin	++
Inulin	−
Galacto-oligosaccharides	+++
Glycogen	−
Amylopectin	−

A minus sign (−) indicates that final OD_600_ < 0·3, (+) indicates final OD_600nm_ = 0.3–0.5, (++) indicates final OD_600nm_ = 0.5–0.8 and (+++) indicates final OD_600nm_ > 0.8. Experiments were performed in triplicate.

## Supplementary Materials

The following are available online at https://www.mdpi.com/2076-2607/8/9/1313/s1. Figure S1. LGI and WAS models. Figure S2. Dotplot alignments of fully sequenced *B. bifidum* genomes.
