# Supplementary material for: The Infant-Derived Bifidobacterium bifidum Strain CNCM I-4319 Strengthens Gut Functionality
Source: Microorganisms. 2020 Aug 28;8(9):1313. doi: 10.3390/microorganisms8091313 (PMC7565306; doi:10.3390/microorganisms8091313)
Supplement: Supplementary file 1 [file microorganisms-08-01313-s001.pdf]

microorganisms

Article

The Infant-Derived *Bifidobacterium bifidum* Strain CNCM I-4319 Strengthens Gut Functionality

Rebeca Martín [1,†], Francesca Bottacini [2,†], Muireann Egan [2], Celia Chamignon [1], Valérie Tondereau [3], Raphaël Moriez [4], Jan Knol [5,6], Philippe Langella [1], Hélène Eutamene [3], Tamara Smokvina [4,*] and Douwe van Sinderen [1,7,*]

1 Commensal and Probiotics-Host Interactions Laboratory, Micalis Institute, INRA, AgroParisTech, Université Paris-Saclay, 91190 Jouy-en-Josas, France; rebeca.martin-rosique@inrae.fr (R.M.); celia.chamignon@inrae.fr (C.C.); philippe.langella@inrae.fr (P.L.)
2 APC Microbiome Ireland, University College Cork, Cork T12 K8AF, Ireland; f.bottacini@umail.ucc.ie (F.B.); muireann.egan@umail.ucc.ie (M.E.)
3 Neurogastroenterology & Nutrition Group, Toxalim INRAE, Université de Toulouse, ENVT, INP-Purpan, 31058 Toulouse, France; valerie.tondereau@inrae.fr (V.T.); helene.eutamene@inra.fr (H.E.)
4 Danone Nutricia Research, 91767 Palaiseau, France; raphael.moriez@danone.com
5 Danone Nutricia Research, 3584 CT Utrecht, The Netherlands; jan.knol@danone.com
6 Laboratory of Microbiology, Wageningen University, 6708 PB Wageningen, The Netherlands
7 School of Microbiology, University College Cork, Cork T12 K8AF, Ireland
* Correspondence: tamara.smokvina@danone.com (T.S.); d.vansinderen@ucc.ie (D.v.S.)
† Joint first author.

Supplementary Material

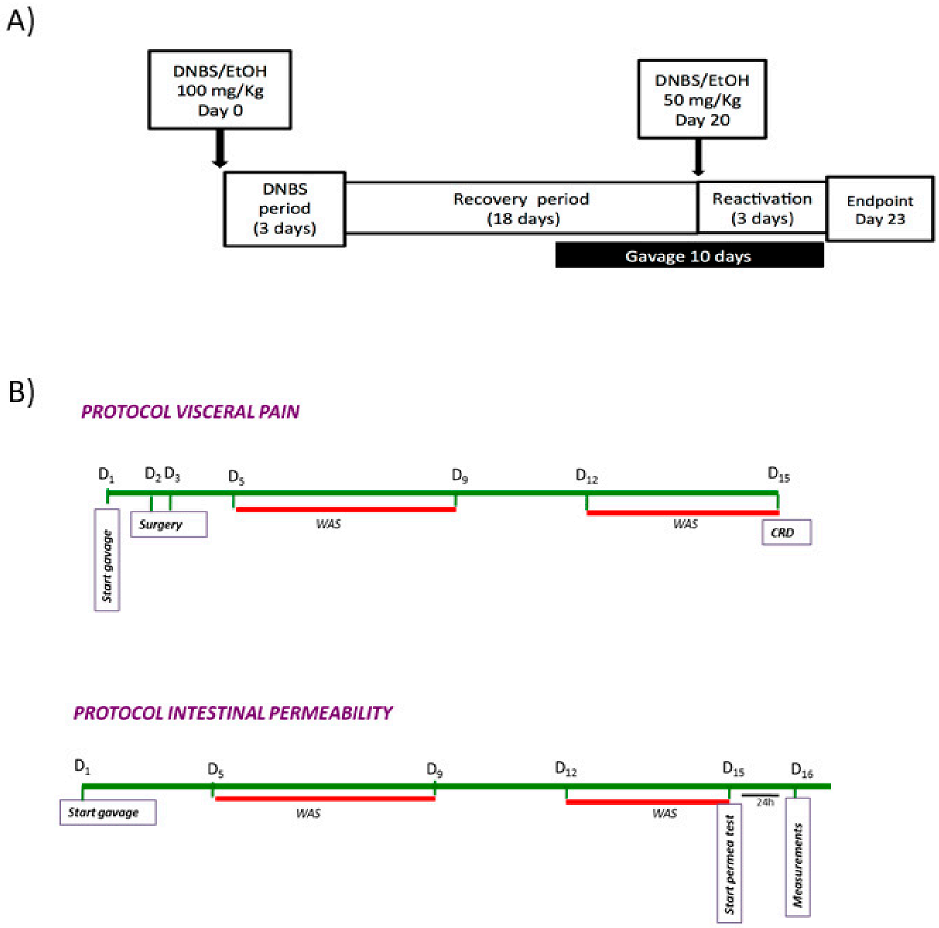

Figure S1. LGI and WAS models. Time course protocols employed for LGI (A) and WAS (B) experiments. See Materials and Methods section for details on these protocols.

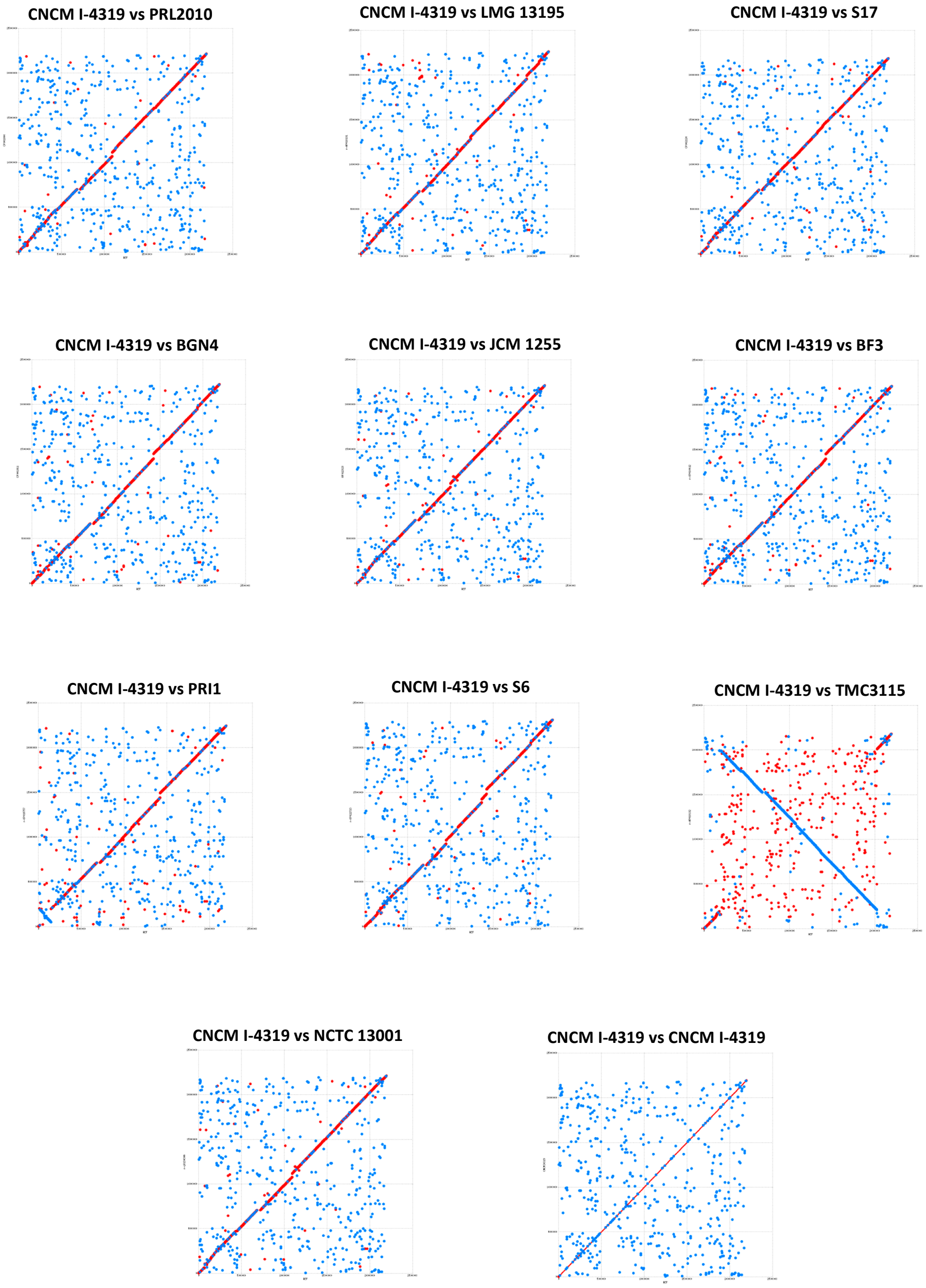

Figure S2. Dotplot alignments of fully sequenced *B. bifidum* genomes. Dotplot alignment showing the sequence synteny of *B. bifidum* CNCM I-4319 and 10 publicly available and fully sequenced *B. bifidum* genomes.
